# Involvement of *Daphnia pulicaria* Sir2 in regulating stress response and lifespan

**DOI:** 10.18632/aging.100909

**Published:** 2016-02-29

**Authors:** Charles A. Schumpert, Craig Anderson, Jeffry L. Dudycha, Rekha C. Patel

**Affiliations:** ^1^ Department of Biological Sciences, University of South Carolina, Columbia, SC 29208, USA

**Keywords:** Daphnia, Sir2, heat shock, lifespan, RNAi

## Abstract

The ability to appropriately respond to proteotoxic stimuli is a major determinant of longevity and involves induction of various heat shock response (HSR) genes, which are essential to cope with cellular and organismal insults throughout lifespan. The activity of NAD+-dependent deacetylase Sir2, originally discovered in yeast, is known to be essential for effective HSR and longevity. Our previous work on HSR in *Daphnia pulicaria* indicated a drastic reduction of the HSR in older organisms. In this report we investigate the role of Sir2 in regulating HSR during the lifespan of *D. pulicaria*. We cloned *Daphnia* Sir2 open reading frame (ORF) to characterize the enzyme activity and confirmed that the overall function of Sir2 was conserved in *Daphnia*. The Sir2 mRNA levels increased while the enzyme activity declined with age and considering that Sir2 activity regulates HSR, this explains the previously observed age-dependent decline in HSR. Finally, we tested the effect of Sir2 knockdown throughout adult life by using our new RNA interference (RNAi) method by feeding. Sir2 knockdown severely reduced both the median lifespan as well as significantly increased mortality following heat shock. Our study provides the first characterization and functional study of *Daphnia* Sir2.

## INTRODUCTION

The ability to respond to proteotoxic stress has proven to be a key regulator in the aging process in several model organisms [1, 2]. Aging is a universal biological process that leads to a decline in multiple functions at the organismal and cellular levels [3-5]. Although several pathways responsible for the aging process have been described, the crosstalk between such regulatory pathways is not fully understood. Aging is an unavoidable biological process that is a culmination of multiple cellular pathways being rendered dysfunctional over time [5]. One well-studied mechanism that regulates the aging process involves cellular responses to proteotoxic stress, also referred to as proteostasis [2, 6]. Clancy and Birdsall, among others, attribute the pathologies and phenotypes associated with aging to the loss of proteostasis and the resulting inability to respond to various proteotoxic damages [6, 7]. The predominant cellular response to proteotoxic stimuli is the heat shock response (HSR), which has been studied extensively in numerous species [1]. The HSR involves the induction of molecular chaperones, termed heat shock proteins (Hsps) for relieving the molecular damage. The Hsps either renature the misfolded or damaged proteins following stress or target them for degradation in case the damage is too severe [1, 2]. Heat shock protein 70 (Hsp70) is one of the main Hsps responsible for such remedial action [8]. Following a proteotoxic insult (heat shock, exposure to heavy metals, oxidative stress, or exposure to extreme pH), Hsp70 is induced transcriptionally by the transcription factor heat shock factor (HSF) [1, 9]. Upon heat shock or another proteotoxic event HSF undergoes trimerization and binds to specific sequences within the Hsp70 promoter to induce rapid and robust transcription to combat proteotoxic stress [1].

In multiple model organisms, previous studies have established that the HSR declines with age [10-12]. Studies in *C. elegans* as well as *D. melanogaster* have demonstrated the inability of old organisms to respond to proteotoxic stress [11, 13]. Previous studies have also demonstrated that the inability to mount a strong HSR is due to the inability of HSF to bind to DNA in old organisms [12, 14, 15]. One mechanism leading to this decline of HSF's DNA-binding activity during aging is attributed to the post-translational modifications of HSF [16, 17]. In order for HSF to trimerize and become active for DNA-binding, HSF has to be deacetylated by the NAD+-dependent histone deacetylase Sir2 (named Sirt1 in mammals, Sir2 in yeast and *Daphnia*). Sir2 was first discovered in yeast and has since been investigated for its role in deacetylation of multiple targets including p53, FoxO, NFκB and HSF [16, 18, 19]. Previous work has also demonstrated that overexpression of Sir2 homologs in yeast, *C. elegans*, and *D. melanogaster* leads to a lifespan extension (30% increase in median lifespan) while a knockdown in Sir2 results in a decrease in lifespan [19-23]. In terms of the HSR, Westerheide *et al* demonstrated that Sirt1 deacetylates HSF1 at K80 and that in WI-38 fibroblasts, expression of Sirt1 declines with passage number or age of fibroblasts [17]. Several studies reported that Sir2/Sirt1 deacetylase activity declines with age, without a corresponding definitive decline in Sir2/Sirt1 protein expression [24-29]. Thus, a decline in HSF's ability to bind to DNA in old organisms may result from inactive, acetylated HSF due to a decrease in Sir2/Sirt1 activity.

We use *Daphnia*, a freshwater microcrustacean, in our studies on aging and lifespan regulation. In particular, we examine the differences in lifespan and aging between two ecotypes *Daphnia pulex* and *Daphnia pulicaria*. These two ecotypes diverged only ∼82,000 years ago with *D. pulicaria* lifespan being more than twice as long as *D. pulex* [30]. The short lived *D. pulex* naturally inhabits small transitory ponds that are found around the world and exhibit a median lifespan of about 20-25 days [31-33]. The closely related, yet long lived *D. pulicaria* inhabits larger, more stable, stratified lakes and has a median lifespan of about 65-70 days [31-33]. *Daphnia* is a useful model organism for research on aging especially due to its unique characteristics [12, 34]. *Daphnia* are easily cultured in the lab and they reproduce via cyclic parthenogenesis making it easy to establish a population of isogenic individuals [35]. The *D*. *pulex* genome is fully sequenced with estimated 30,907 protein coding genes, and has the highest number of genes homologous to the human genome among all sequenced arthropods [36]. Although the list of molecular techniques to make *Daphnia* amenable to molecular studies is still growing, multiple techniques have been established including an RNA interference system, and a gene replacement and targeted mutagenesis system using TALEN and CRISPR/Cas9 systems [37-41].

We have previously studied the HSR of *D. pulex* and *D. pulicaria* in relation to aging [12]. Our results showed that the short-lived *D. pulex* stop responding to proteotoxic stress by middle age whereas the long-lived *D. pulicaria* can still mount a strong HSR at an equivalent age. In both ecotypes, the ability to respond to proteotoxic stimuli was abrogated at old age [12]. We further investigated the possible mechanism for this decline in the HSR and found that although the HSF protein levels were equal throughout lifespan, its ability to bind DNA in old *D. pulicaria* declined [12]. Due to the established role of Sir2 in activation of HSF and the known decrease in its enzymatic activity with age in other organisms, we wanted to investigate the potential role of *Daphnia* Sir2 in regulation of HSR and longevity in *D. pulicaria*.

In the present study, we cloned *D*. *pulicaria* Sir2 open reading frame (ORF), examined Sir2 transcript and activity levels during *D. pulicaria* lifespan, and investigated Sir2's functional role in HSR and lifespan regulation by performing gene-specific RNA interference (RNAi). We demonstrate that Sir2 ORF cloned from *D. pulicaria* (Clone: LakeXVI-11) produces a functional protein that has similar overall functions to mammalian Sirt1. Cell viability experiments examining the effects of *Daphnia* Sir2 overexpression following a severe heat shock showed that similar to mammalian Sirt1, *Daphnia* Sir2 confers a protective effect resulting in a markedly reduced cell death following proteotoxic stress. *Daphnia* Sir2 overexpression in mammalian cells also exhibits an enhanced HSR as measured by a transcriptional reporter assay. Although the transcript levels for Sir2 increased with age in *D. pulicaria*, the enzymatic activity of Sir2 showed significant decrease with age. Finally, using our newly developed RNAi via feeding system we knocked down Sir2 in adult *Daphnia* throughout their lifespan. A knockdown of Sir2 expression in adult *Daphnia*, led to a drastic shortening of lifespan by 64 +/− 2% compared to wild-type control organisms. This is the first report demonstrating a decrease in *Daphnia* lifespan as a result of a targeted knockdown of an established longevity gene by using RNAi via feeding method.

## RESULTS

### *Daphnia* Sir2 protein shows sequence conservation of residues essential for its enzyme activity

**Figure 1:**
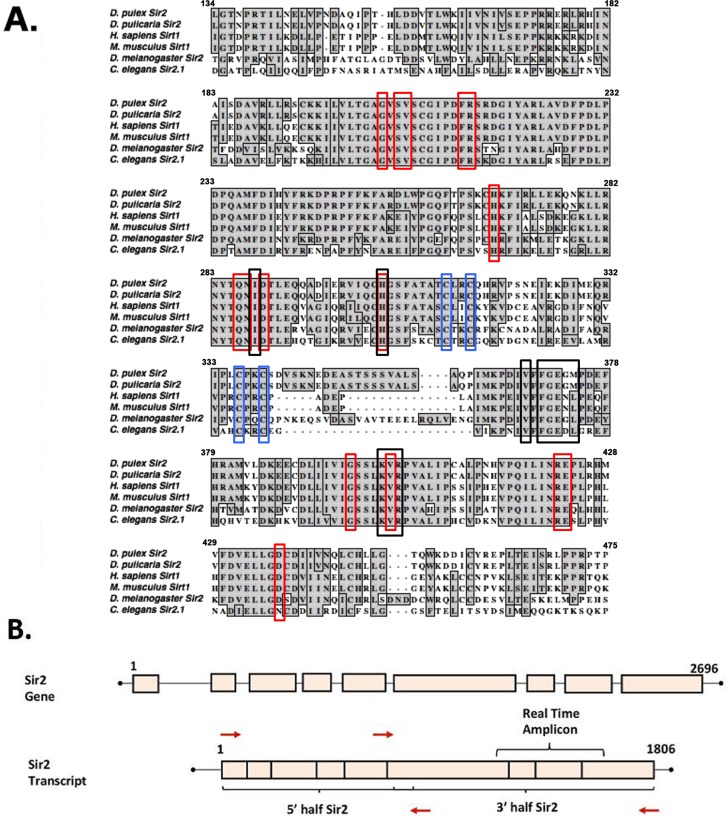
Sequence alignment of the Sirt1 homologs from invertebrates and vertebrates **(A)** Sir2 related proteins from 5 model organisms are aligned with *Daphnia pulex* Sir2 on the top. Red boxes depict residues involved in NAD+ binding, black boxes depict substrate-binding residues, and blue boxes depict zinc-binding residues. Alignments made using ClustalW in MacVector. The accession numbers of the sequences used are: *Daphnia pulex* Sir2: DappuDraft_290541 (wFleaBase), *Daphnia pulicaria* Sir2: KT960973, *H. sapiens* SIRT1: NP_036370.2, *C. elegans* SIR2.1: NP_001255484.1, *D. melanogaster* Sir2: AAC79684.1, and *M. musculus* SIRT1: NP_062786.1. **(B)** Schematic representation of *Daphnia* Sir2 gene and Sir2 transcript. PCR primers (red arrows) were designed such that the open reading frame (ORF) was PCR amplified in two parts, a 5′ half and a 3′ half as indicated. Real-time amplicon is the region of the transcript amplified via real time PCR for expression analysis.

The *D. pulex* genome contains five homologs in the sirtuin gene family and we wanted to assess sequence conservation in the essential catalytic domain of Sir2, the one-to-one ortholog of human Sirt1. Such conservation would be indicative of similar catalytic protein deacetylase activity of the *Daphnia* protein. Thus, we aligned the Sir2 protein sequence from the published *D*. *pulex* genome with previously studied Sir2/Sirt1 sequences from other organisms. As is shown in figure 1A, there is a high degree of sequence similarity between the catalytic domain of *Daphnia* Sir2 and the sequences from *Drosophila*, human, mouse, and worms. Of particular note, the residues that exhibit the highest conservation include those from the domains of the protein essential for the deacetylase enzyme functions such as NAD+ and substrate binding. The alignment in figure 1A shows the part of *D. pulex* Sir2 that exhibits the highest homology with the corresponding regions from other organisms known to be required for the catalytic function of the Sir2 enzyme.

### Characterization of *Daphnia* Sir2

**Figure 2:**
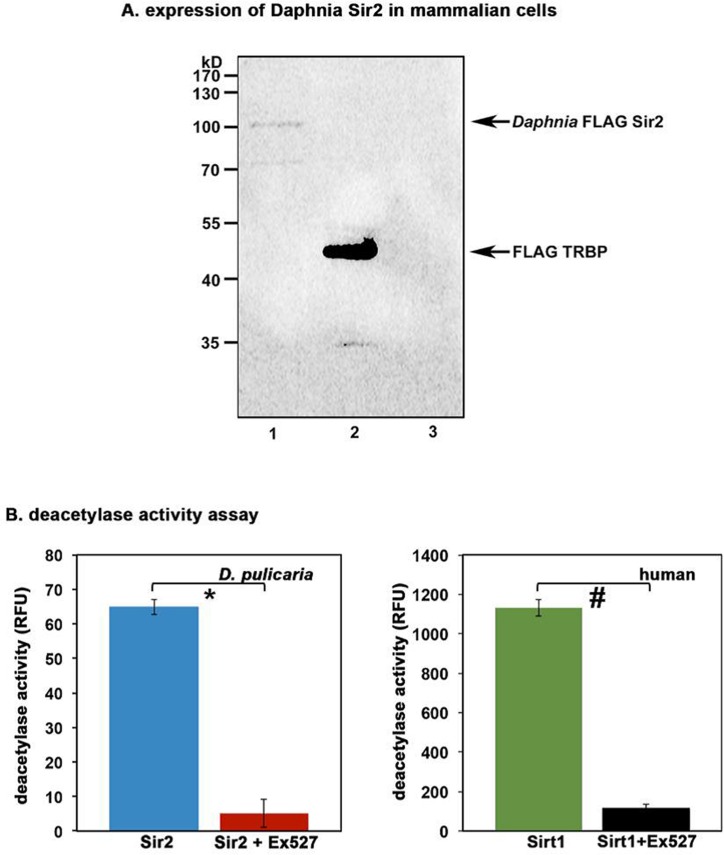
Daphnia Sir2 ORF produces a functional protein with catalytic activity **(A) Expression of *Daphnia* Sir2 in Cos-1 cells**. 100 ng each of *Daphnia* FLAG-Sir2/pcDNA3.1-, FLAG-TRBP/pcDNA3.1-, or empty vector pcDNA3.1- were transfected in Cos-1 cells using Effectene (Qiagen) and total protein extract was prepared 24 h after transfection. Western blot analysis was performed with anti-Flag antibody (M2, Sigma). Arrows indicate Flag-Sir2 and Flag-TRBP positions. **(B) Sirt1 Activity Assay**. Activity assay was performed using 5 μl of *in vitro* translated *Daphnia* Sir2. Human recombinant Sirt1 was used as a positive control. Ex527, a Sirt1/Sir2 specific inhibitor was used to ensure specificity that the observed deacetylase activity was that of *Daphnia* Sir2. Note that *Daphnia* Sir2 data represented is after subtracting the background activity obtained with unprimed (no plasmid DNA added) reticulocyte lysate. Error bars indicate standard deviations from 4 replicate assays. A Student's T Test was performed to determine statistical significance. The p values are as follows: *: 5.2×10^−7^ (t stat: 22.2, df: 3); #: 4.8×10^−7^ (t stat: 22.6, df: 3).

Using PCR to amplify the open reading frame (ORF) in two overlapping pieces as outlined in Figure 1 B, we first obtained the 5′ and 3′ halves of *D. pulicaria* Sir2 ORF. After verification of the sequence of the PCR products, using the internal restriction enzyme MscI, we joined the two halves as outlined in methods section to create a complete ORF that would be capable of coding for the entire Sir2 protein. We sub-cloned the Sir2 ORF in a mammalian expression vector pcDNA3.1- (Invitrogen) with a Flag tag on its N-terminus so we could check its expression in cell culture as well as in an *in vitro* transcription–translation system to obtain a full-length Sir2 protein for measuring its catalytic activity. Thus, in order to determine if the cloned *Daphnia* ORF would produce a functional enzyme, we performed two experiments, one to determine the expression of Sir2 in mammalian cells and another to ensure enzymatic activity of the Sir2 produced by *in vitro* translation of the ORF. Currently there are no *Daphnia* or other crustacean cell lines available, thus we used mammalian cells to express *Daphnia* Sir2. Figure 2A depicts a western blot analysis of the protein extract from Cos-1 cells transfected with *Daphnia* FLAG-Sir2/pcDNA3.1- expression construct or with a positive control FLAG-TRBP/pcDNA3.1-. The FLAG-TRBP/pcDNA3.1- serves as a positive control for the transfection, expression, and western blot as we have used this plasmid for many studies previously [42, 43]. As seen in lane 1, the band just above the 100 kD molecular weight marker corresponds to *Daphnia* FLAG-Sir2. The calculated molecular weight of *Daphnia* Sir2 based on its primary sequence is 67kD, however, Sir2 proteins including mammalian Sirt1 are known to run higher on the SDS-PAGE gels than their calculated molecular weight based on the primary sequence, which results from the heavy post-translational modifications. Lane 2 indicates a strong expression of FLAG-TRBP, which is known to express extremely strongly based on our previous work [42, 43]. As expected, there is no band present in lane 3 as it contains extract from Cos-1 cells transfected with empty vector pcDNA3.1-, which will not express Flag-Sir2. Thus, the *Daphnia* Sir2 is expressed in Cos-1 cells and produces a full-length protein.

We next wanted to determine if the protein produced from the *Daphnia* Sir2 ORF has deacetylase activity similar to Sir2 from other organisms. We used the FLAG-Sir2/pcDNA3.1- construct to produce an *in vitro* translated *Daphnia* Sir2 protein using the TNT T7 coupled reticulocyte system (Promega). The protein was synthesized using S^35^ labeled methionine and its production and integrity was checked by SDS-PAGE followed by a phosphorimager scan (data not shown). The deacetylase activity present in the reticulocyte lysate was measured using a Sirt1 Activity Assay kit (Abcam). Shown in Figure 2B, the reticulocyte lysate containing *in vitro* translated *Daphnia* Sir2 exhibits deacetylase activity (blue bar). A human recombinant Sirt1 sample that was supplied with the kit was used as a positive control (green bar). Note that a reticulocyte lysate only control was performed as a negative control with no Sir2/pcDNA3.1- DNA added as this represents the activity present in reticulocyte lysate. The activity obtained from this negative control is subtracted from the values displayed in Figure 2B. The red and the black bars represent the enzyme activity in presence of a Sir2 specific inhibitor Ex527 and establish that both the human recombinant Sirt1 as well as *Daphnia* Sir2 activity is significantly diminished by the inhibitor. These results further confirm that the cloned ORF encodes a functional Sir2 protein.

### *Daphnia* Sir2 protects against proteotoxic stress similar to mammalian Sirt1

**Figure 3:**
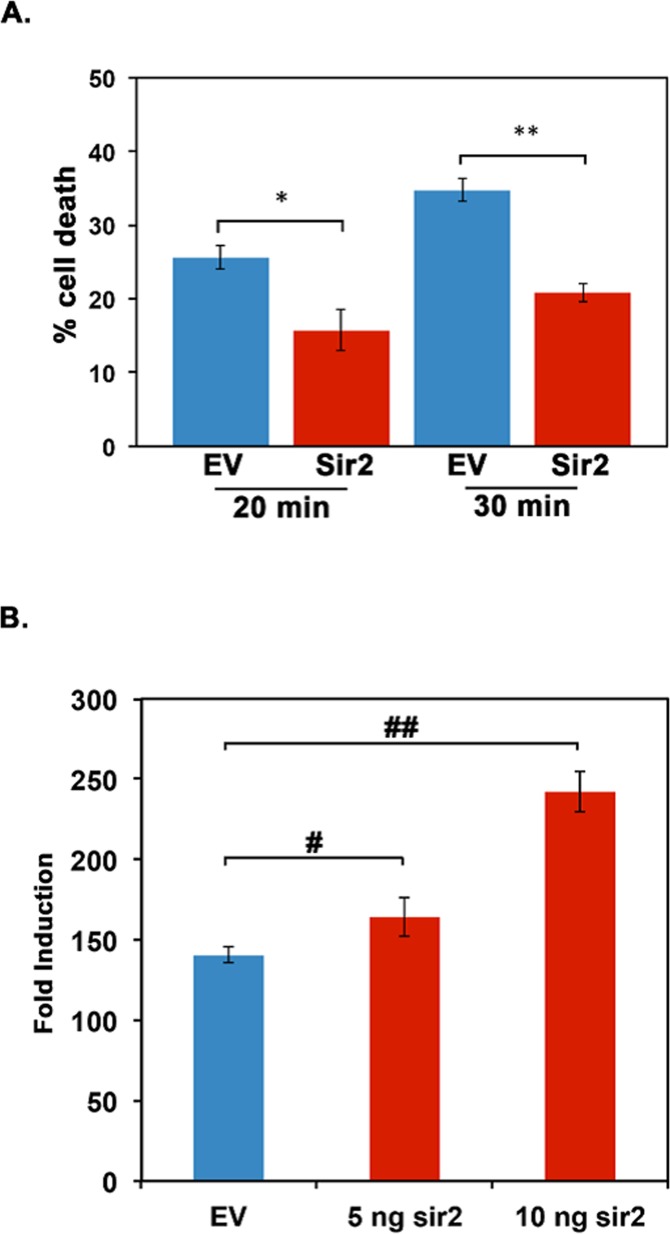
Daphnia Sir2 exhibits functional activity similar to mammalian Sirt1 **(A) Daphnia Sir2 overexpression confers cytoprotection to heat shock.** Cos-1 cells were transfected with *Daphnia* Sir2 expression construct and heat shocked at 42^0^ C for 20 and 30 minutes as indicated. Blue bars depict empty vector control and red bars represent the *Daphnia* Sir2 transfections. Percent cell death was measured using Trypan Blue viability assay 24 hours following heat shock. Error bars represent standard deviations. **(B) *Daphnia* Sir2 enhances the transcriptional induction of genes regulated by a heat shock.** Cos-1 cells were co-transfected either with the empty vector (blue bars) or *Daphnia* Sir2 expression construct (red bars), pGL4.41 (luciferase reporter plasmid, Promega), and pRL–Null (for normalization of transfection efficiency, Promega) and treated with CdCl_2_ at 24 hours following transfection. The luciferase assay was performed on cell extracts 6 hours following CdCl_2_ treatment. Error bars represent standard deviations. A Student T test of arcsin transformed proportions was used to determine statistical significance in A (* t stat: 3.98, df: 3, p: 0.007; ** t stat: 7.37, df: 3, p: 0.0003). In B an ANOVA was performed (F: 309, df: 2,6, p:9.2×10^−5^) followed by a post hoc Tukey test, both # and ## indicate differences between the means at p < 0.5.

In order to verify the functional activity of *Daphnia* Sir2, we tested if *Daphnia* Sir2 would offer protection against heat shock in a mammalian cell culture system. Previous work on mammalian Sirt1 in 293T cells established that Sirt1 overexpression provided protection to the cells during severe heat shock [17]. To assess if *Daphnia* Sir2 overexpression would offer similar protection, we transfected Cos-1 cells with the *Daphnia* Sir2 expression construct and measured the cell viability following a severe heat shock. Shown in Figure 3A, *Daphnia* Sir2 overexpression in Cos-1 cells resulted in lower percentage cell death following a heat shock. With 20 minutes of heat shock, there was 25.67 +/− 1.62% cell death in cells transfected with empty vector without any Sir2 ORF insert (blue bar). In contrast to this, only 15.74 +/− 2.74% cell death was observed with Sir2 overexpression construct (red bar). When duration of heat shock was 30 minutes, the differences in percentage was greater with control being at 34.79 +/− 1.63% cell death (blue bar) and Sir2 overexpression sample showing 20.90 +/− 1.27% cell death (red bar). Thus, *Daphnia* Sir2 overexpression is clearly protective to mammalian cells following heat shock, potentially acting via the deacetylation of the transcription factor HSF1. The results also indicate that *Daphnia* Sir2 has an enzymatic activity similar to *Drosophila* Sir2 and mammalian Sirt1.

In order to further analyze *Daphnia* Sir2's potential in regulating response to proteotoxic stress, we performed co-transfection experiments in Cos-1 cells using *Daphnia* Sir2 expression construct and a luciferase reporter pGL4.41 (Promega), which is regulated transcriptionally by proteotoxic stress. pGL4.41 contains four mammalian heat shock elements (HSEs) that serve as binding sites for HSF1 [1] to induce the transcription of the downstream reporter firefly luciferase ORF only when proteotoxic stress (heat shock or heavy metals) is present. Thus, under proteotoxic conditions, there is an induction of luciferase activity via the activation and binding of HSF1 to the HSEs in pGL4.41. We co-transfected the *Daphnia* Sir2 expression construct and pGL4.41 and treated cells with cadmium chloride (CdCl_2_) to induce proteotoxic stress. As shown in Figure 3B, overexpression of *Daphnia* Sir2 caused a dose-dependent increase in luciferase activity after CdCl_2_ treatment as compared to empty vector control where no *Daphnia* Sir2 was present. The empty vector control (blue bar) showed a 140.50 +/− 4.95 fold induction in response to CdCl_2_. Co-transfection of 5 ng and 10 ng of *Daphnia* Sir2 expression construct resulted in 164.16 +/− 11.9, and 242.18 +/− 12.72 -fold induction respectively (red bars). Thus, *Daphnia* Sir2 overexpression in a mammalian cell culture system results in a more robust cellular response to proteotoxic stress, potentially via the deacetylation of HSF1.

### *Daphnia* Sir2 mRNA levels increase with age in *D. pulicaria*

**Figure 4:**
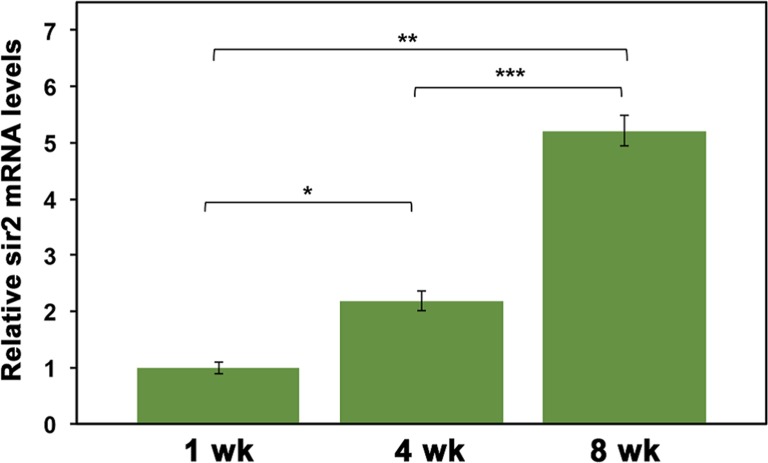
Sir2 mRNA levels increase with *D. pulicaria* age Total RNA was harvested from groups of 15 *D. pulicaria* at each age. Real-time PCR was performed using a BioRad CFX 96 using Sir2-specific primers and GAPDH primers as a normalization control. Data was analyzed using the 2^−ΔΔCt^ method and are represented as relative Sir2 mRNA levels (green bars). Error bars represent standard deviations. The ages are as indicated, wk: weeks. To analyze statistical significance, an ANOVA was performed (F: 145.95, df: 2,6, p:1.38×10^−7^) followed by a post hoc Tukey test. The post hoc analysis revealed that all three means were statistically different from one another and for all: * (statistical significance between 1 wk and 4 wk), ** (statistical significance between 1 wk and 8 wk), and *** (statistical significance between 4 wk and 8 wk) and indicate p<0.05.

The expression of Hsp70 in response to a heat shock decreases with increasing age in *D. pulicaria* [12], which may be due to a decreased expression of Sir2. In order to analyze the expression of Sir2 during the lifespan of *D. pulicaria*, we examined the Sir2 transcript levels at ages 1, 4, and 8 weeks. Total RNA was isolated, converted to cDNA, and real time PCR was performed to examine the relative amount of Sir2 mRNA. As represented in Figure 4, D*. pulicaria* steady state Sir2 mRNA levels increased with age. These results indicate that decreased expression of Sir2 mRNA is not a likely cause of decreased HSR and reduced Hsp70 expression in response to proteotoxic stress in old organisms.

**Figure 5:**
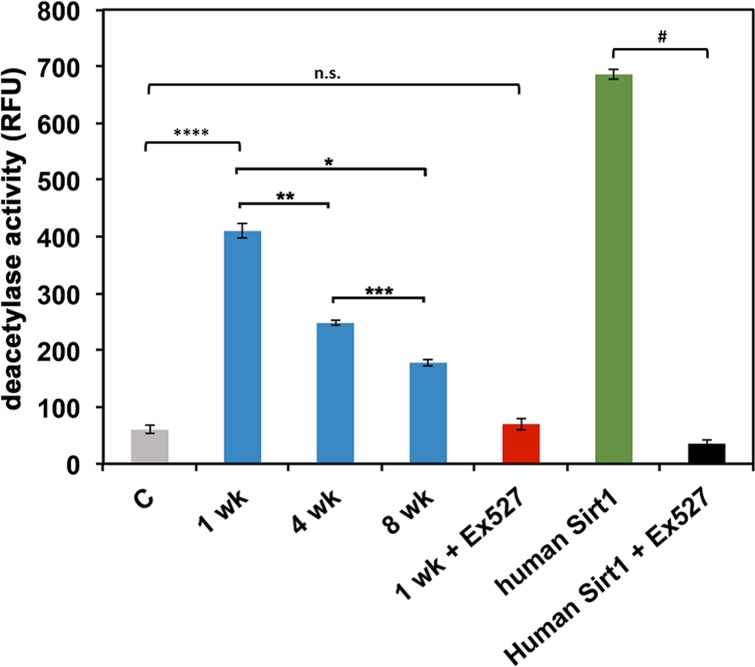
D. pulicaria Sir2 activity declines with age The deacetylase activity present in total protein extracts was assayed using a Sirt1 activity assay kit (Abcam). *Daphnia* extracts (1 μg total protein) prepared from the indicated ages (wk: weeks) were used (blue bars). NAD+ was added in excess (non-limiting amounts) to the assay mixtures. Recombinant human Sirt1 (Abcam) was used as a positive control (green bar; 30 ng protein). Ex527, a Sirt1/Sir2 specific inhibitor (800 nM), was utilized to ascertain that the deacetylase activity was from Sir2 and not another sirtuin (red bar for *Daphnia* Sir2, aged 1 week; black bar for human recombinant Sirt1, both 1 μg protein). A negative control with no extract added is represented as a gray bar. Error bars represent standard deviations. A Student T-test was performed to examine statistical significance between human Sirt1 and human Sirt1 plus Ex527: # t stat: 563.4, df: 5, p: 3.3×10^−13^. An ANOVA was performed (F: 911, df: 4,10, p: 9.2×10^−13^) followed by a post hoc Tukey test. The post hoc analysis revealed that all means were statistically different from one another for all: * (statistical significance between 1 wk and 8 wk), ** (statistical significance between 1 wk and 4 wk), *** (statistical significance between 4 wk and 8 wk), **** (statistical significance between no extract control and 1 wk) and indicate p<0.05, except between control and 1 wk plus Ex527, N.S.: not significant.

We next examined if Sir2 enzyme activity declines with age in *D. pulicaria*. Sir2 activity assays were performed using a commercially available kit (Abcam). As represented in Figure 5, Sir2 activity of *D. pulicaria* declines with age, 1 wk old organisms displayed the highest activity of Sir2 (410.0 +/− 12.02 RFUs, first blue bar from the left), followed by 4 wk old *D. pulicaria* (248.6 +/− 4.36 RFUs, second blue bar from the left) and the 8 wk old organisms had the lowest amount of Sir2 activity (178.2 +/− 5.44 RFUs, third blue bar from the left). To ascertain that we were measuring Sir2 activity, we tested the enzyme activity in presence of Ex-527, a known specific inhibitor for Sirt1. As seen in figure 5 (1 wk + Ex527; red bar and human Sirt1 + Ex527; black bar), the deacetylase activity drops to background levels (grey bar labeled C) in the presence of 800 nM Ex527. Thus, it can be concluded that the commercially available assay system works efficiently to assay *Daphnia* Sir2 activity and that Sir2 activity declines with age in *D. pulicaria*.

### Targeted knockdown of Sir2 expression via RNAi shortens *D. pulicaria* lifespan and enhances death following proteotoxic stress

**Figure 6:**
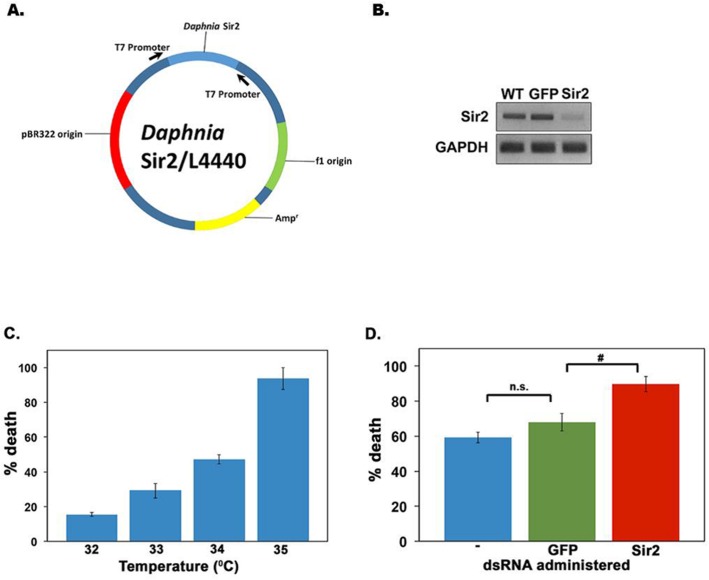
Targeted RNAi knockdown of D. pulicaria Sir2 severely impairs HSR and survival **(A) Diagram of Sir2 targeting construct. (B) Sir2 transcript levels are diminished following an RNAi feeding regimen of bacteria expressing sir2 dsRNA**. Total RNA was isolated from untreated, GFP dsRNA treated, or Sir2 dsRNA-treated *Daphnia* after the RNAi feeding regimen for ten days. Reverse transcriptase-PCR was performed for 27 cycles with Sir2 or GAPDH specific primers and the product was analyzed on a 1% agarose gel. **(C) Mean percentage death following a heat shock in *D. pulicaria*.** *D. pulicaria* were heat shocked at various temperatures as indicated for 30 minutes, allowed to recover for 24 hours, at which point the percentage death was measured. Error bars represent standard deviations. **(D) Mean percentage death in response to heat shock following Sir2 knockdown**. After ten days on an RNAi feeding regimen, untreated *D. pulicaria* (labeled -; blue bar), GFP dsRNA treated *D. pulicaria* (green bar), and Sir2 dsRNA treated *D. pulicaria* (red bar) were subjected to heat shock at 34^0^ C for 30 minutes, allowed to recover for 24 hours, and the percentage death was measured. We performed RNAi in a total of 30 individuals per treatment group (for a total of 90 individuals in the experiment) over the course of 4 biological replicates. Error bars represent standard deviations. An ANOVA was performed (F: 146, df: 2,9, p:1.35×10^−7^) followed by a post hoc Tukey test, # indicate differences between the means at p < 0.5.n.s.: not significant.

A decline in Sir2 activity has been shown to be responsible for aging phenotypes of various model organisms [44]. In addition, evidence exists for extension of lifespan by enhancing Sir2 activity with nutraceuticals or by overexpression in model organisms [19, 44]. Thus, we next examined if a targeted knockdown of Sir2 activity in *D. pulicaria* would shorten the lifespan. We recently developed an effective RNAi method for adult *Daphnia* via feeding. In order to achieve RNAi for the gene of interest, a double-stranded RNA (dsRNA) corresponding to a selected region in the target mRNA is expressed in *E.coli* using the L4440 vector [41, 45]. *Daphnia* are fed on these bacteria to deliver the dsRNA systemically via the gut. Using this method we have previously achieved effective RNAi of phenoloxidase expression in *D. melanica* and eyeless expression in *D. melanica* and *D. pulicaria* [41], unpublished). Figure 6A shows the map of the targeting construct for *Daphnia* Sir2 using the first 863 bp of the Sir2 transcript. We used ten *D. pulicaria* per beaker in 100 ml of COMBO for feeding them the bacteria that expressed Sir2 or GFP dsRNA for 10 days, after which we checked if gene–specific RNAi was achieved. To ensure that the Sir2 specific RNAi was achieved, we analyzed the steady state Sir2 mRNA levels after the RNAi feeding regimen. Figure 6B displays data from a representative reverse transcriptase PCR demonstrating a knockdown in Sir2 mRNA only in RNA isolated from *D. pulicaria* fed on Sir2 dsRNA expressing bacteria (lane 3). RNA isolated from *D. pulicaria* fed on bacteria expressing GFP dsRNA (lane 2) and wild-type *D. pulicaria* controls not fed on bacteria (lane 1) both have constitutive levels of Sir2 mRNA expression with little variation in Sir2 transcript levels. Thus, our targeting Sir2 dsRNA construct achieved very effective and specific Sir2 knockdown. In order to measure the effect of Sir2 knockdown on viability of *Daphnia* after a heat shock, we first determined the optimal heat shock conditions. We subjected *D. pulicaria* to a range of elevated temperatures as indicated in figure 6C and measured percentage death 24h after heat shock. Based on these results we selected 34^0^ C as the heat shock temperature, as it would allow us to measure an increase or a decrease in lethality after Sir2 knockdown. To analyze the effect of Sir2 knockdown on the HSR, *Daphnia* that were fed no bacteria (blue bar labeled “– “, Figure 6D), bacteria with a control construct (encoding GFP dsRNA- green bar, Figure 6D), or bacteria with the Sir2 targeting construct (encoding Sir2 dsRNA- red bar, Figure 6D) for ten days were heat shocked at 34°C for 30 minutes. As seen in Figure 6D, the *Daphnia* fed on bacteria with sir2 dsRNA had a significantly higher percentage of death following heat shock (red bar: 90 +/− 4.3%), compared to the negative control GFP (green bar: 67 +/− 5.0%), and wild type *D. pulicaria* (blue bar: 59 +/− 3%). Thus, when Sir2 is knocked down in *D. pulicaria*, the organism no longer responds appropriately to proteotoxic stress leading to death following a heat shock.

**Figure 7:**
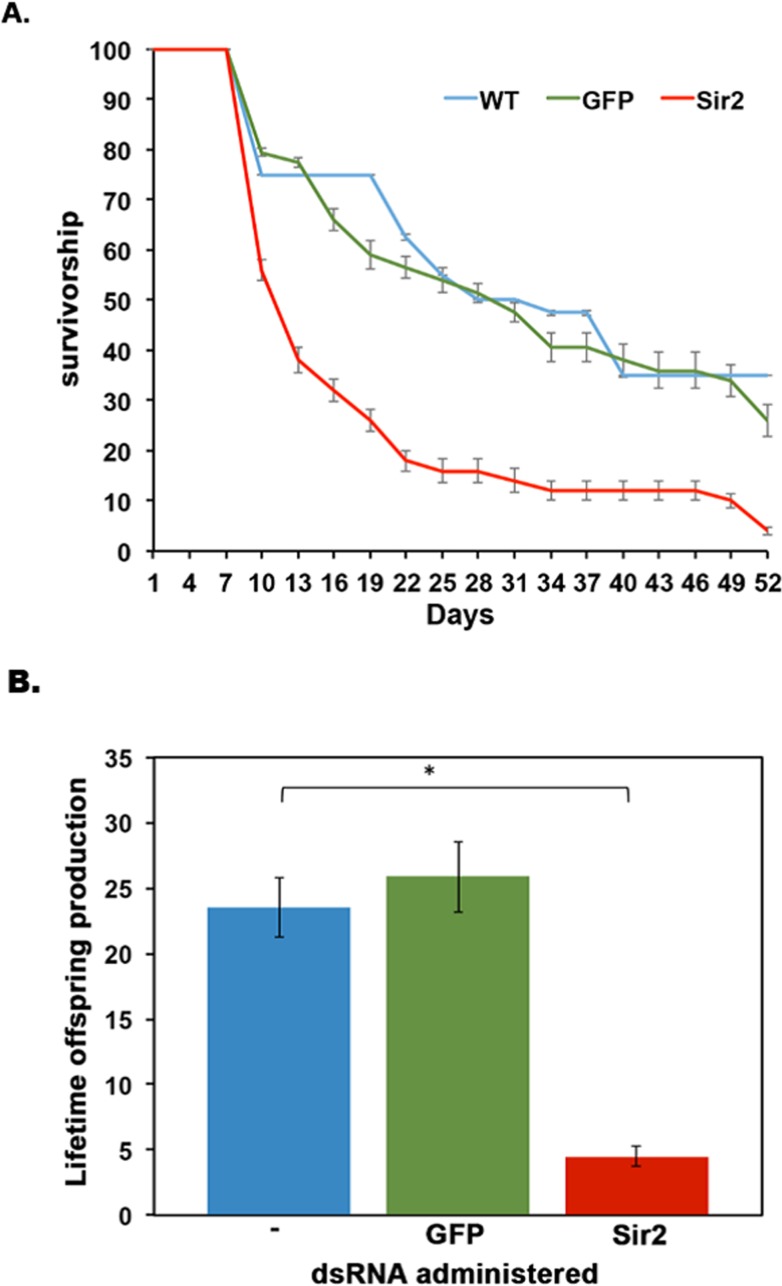
Sir2 knockdown in D. pulicaria results in reduced lifespan and lifetime offspring production **(A) Survivorship curves**. The survivorship curves of untreated (blue line), GFP dsRNA-treated (green line), and Sir2 dsRNA-treated (red line) *Daphnia* are represented. Ten *Daphnia* placed in 100 ml of COMBO were fed daily on bacteria containing the knockdown constructs. RNAi was performed in a total of 4 different biological replicates. Each plotted point is average survivorship on that day (collectively from our 4 biological replicates) and error bars depict standard error of the mean. No bacteria (untreated) (n=40), GFP (dsRNA control) (n=44), Sir2 (n=60). To determine statistical significance, a nonparametric log rank test was performed and the results are as follows: X^2^=19.1, p-value=1.2×10^−5^ comparing Sir2 to WT, X^2^=18.098, p-value=2.1×10^−5^ comparing Sir2 to GFP. **(B) Average number of offspring per *Daphnia* mother.** The average number of progeny per mother over the course of its lifespan was calculated by dividing the total number of offspring produced in an experimental set by the total number of adult *Daphnia* mothers in that set. Blue bar is WT control, green bar is GFP dsRNA-treated, and red bar is Sir2 dsRNA-treated. A Student T-test was performed on the lifetime offspring production and the associated p-values are as follows: *:0.015. Error bars represent standard deviations. An ANOVA was performed (F: 93, df: 2,6, p: 3.02×10^−5^) followed by a post hoc Tukey test. The post hoc analysis revealed that – and Sir2 means were statistically different from one another as are GFP to Sir2, * indicate p<0.05. Means – to GFP were not statistically significant in their difference (ns).

In addition to the deleterious effect of Sir2 knockdown on HSR, we further analyzed the effect of Sir2 knock down on the lifespan of *D. pulicaria*. Sir2 knockdown or mutation has been shown to cause a decline in lifespan for numerous organisms, including yeast, *C. elegans*, and *D. melanogaster* [21-23]. We used ten *Daphnia* neonates per beaker in 100 ml of COMBO and subjected them to an RNAi feeding regimen as described in methods. We followed these *Daphnia* throughout life, feeding them daily with either no bacteria (WT, control), bacteria expressing GFP dsRNA, or bacteria expressing Sir2 dsRNA. The water was changed every other day and reproduction and death was noted for each treatment group. In Figure 7A, the survivorship curves of *Daphnia* on the RNAi feeding regimens are displayed. WT *Daphnia* fed just algae throughout the course of the experiment lived the longest with the median lifespan being 31 +/− 2 days (blue line). The *Daphnia* fed on bacteria expressing GFP dsRNA had a median lifespan of 29 +/− 1.8 days (green line) and the *Daphnia* fed on bacteria expressing Sir2 dsRNA had a median lifespan of only 11 +/− 2.5 days (red line). Note that juvenile mortality was removed from this experiment to examine the overall lifespan effects of Sir2 knockdown on adult *Daphnia*. Overall, a knockdown of Sir2 by RNAi resulted in a 64 +/− 2% reduction in lifespan of *D. pulicaria* compared to WT *D. pulicaria.* These results are statistically significant as analyzed by a nonparametric log rank test (X^2^=19.1, p-value=1.2×10^−5^ comparing WT to Sir2 dsRNA fed sample, X^2^=18.098, p-value=2.1×10^−5^ comparing GFP dsRNA fed sample to Sir2 dsRNA fed sample). The lifetime offspring production was also drastically different for the three groups (Figure 7B) with WT producing an average 20.05 +/− 2.26 offspring per individual (blue bar), GFP dsRNA treated with 25.85 +/− 2.73 offspring per individual (green bar) and Sir2 dsRNA treated *Daphnia* only producing 4.5 +/− 0.75 offspring per individual (red bar). These results establish that Sir2 activity is required for maintaining survival and reproduction in addition to being required for an efficient protective response to proteotoxicity (Figure 6). This is the first report that uses RNAi to examine the effects of the knockdown of a longevity gene in the emerging model system of *Daphnia*.

## DISCUSSION

The longevity factor and deacetylase Sir2/Sirt1 plays a central role in coordinating cellular stress response including HSR and thus affects an organism's ability to respond effectively and appropriately to proteotoxic stress, especially in the context of aging [16, 46]. One of the deacetylation targets for human Sirt1 is HSF1, which is the transcription factor responsible for inducing the HSR [17], and only the deacetylated form of HSF1 is capable of binding to DNA. The age dependent decline in HSR of an organism is one of the factors thought to be responsible for cellular and organismal aging as it contributes to an increased accumulation of misfolded or damaged proteins [2, 47]. Correspondingly, Sirt1 enzyme activity also declines with age in mammals and it is established to be the causative mechanism for the consequent decline in the HSR via inactivation of HSF1 [17, 46].

We have previously established that the HSR declines with age in *Daphnia* similar to mammals and this decline is due to the lack of HSF's ability to bind to DNA [12]. We hypothesized that *Daphnia* Sir2 may regulate HSF's DNA-binding by deacetylation. Although *Daphnia* is an emerging model for research on aging mechanisms, there are very few molecular studies performed with *Daphnia* and aging [12, 34]. In order to provide an initial characterization of the molecular mechanisms known to be operative during the aging process in other model organisms, we tested if *Daphnia* Sir2 has deacetylase activity and if it functions to enhance HSR and lifespan. We cloned Sir2 ORF, tested the activity of encoded protein, and determined the expression levels of Sir2 during the *Daphnia* lifespan. More importantly, we examined the effects of Sir2 knockdown using our newly developed method for RNAi on the lifespan as well as on HSR. Based on our data, *Daphnia* Sir2 offers cellular protection to proteotoxicity by regulating HSR and also functions in regulation of lifespan and reproduction. These results establish the involvement of *Daphnia* Sir2 in HSR, lifespan regulation, and reproduction for the first time.

Regulation of Sir2/Sirt1 activity is complex and is known to occur at multiple levels in mammals and invertebrates [18, 46]. Previous studies have demonstrated that Sirt1 in mammalian cells is regulated by numerous factors including association with activators and inhibitors of catalytic activity, post-translational modifications, as well as transcriptional regulation via transcription factors p53 and FOXO [18, 46]. While the steady state levels of Sir2 mRNA showed an increase during *Daphnia* lifespan, the Sir2 deacetylase activity declined with age. These results suggest a potential regulation at the post-transcriptional or post-translational levels. Although the post-transcriptional regulation of Sirt1 (or Sir2) expression has not been studied extensively, some evidence of regulation by the mRNA-binding protein HuR in mammalian cells [48], and modulation of its enzymatic activity by protein phosphorylation and methylation is reported [18, 46]. Any contribution of such mechanisms in *Daphnia* remains to be explored in the future. In our studies, we could not investigate the relative protein levels of Sir2 during lifespan in *Daphnia* due to the lack of an antibody that can detect *Daphnia* Sir2 protein and thus such potential modes of regulation may only be investigated in future studies once an antibody is available. In addition, cellular NAD+ levels are known to regulate Sir2 activity during aging [19, 49, 50] and may play a role in the decline of Sir2 activity with age in *D. pulicaria*.

To further investigate the functional role of Sir2 in HSR and lifespan regulation in *Daphnia*, we investigated the consequence of Sir2 knockdown by RNAi in adult *Daphnia*. Our results demonstrate a clear increase in mortality following heat shock in *D. pulicaria* with Sir2 knockdown by RNAi (Figure 6). In addition, the median lifespan was significantly shortened in response to Sir2 RNAi (Figure 7). The GFP dsRNA or potentially the presence of bacteria in food alone seem to cause a less significant marginal effect on *Daphnia* lifespan underscoring the importance of such negative controls. The effect of Sir2 knockdown on *Daphnia* reproduction was striking, as the overall lifetime fecundity was severely diminished in Sir2 knockdown adults (Figure 7). Sir2 is considered to be a metabolic sensor, thus, a potential mechanism for this drastic decrease in fecundity may be due to the organism not being able to effectively gauge the nutrient status of the environment [19, 46]. Our results not only demonstrate the role of Sir2 in HSR, lifespan regulation, and offspring production for the first time but also establish that the newly developed RNAi method can be used in adult *Daphnia* to analyze gene function.

The effect of various small-molecule activators of Sir2 on the overall lifespan of *D. pulicaria* remains to be explored in future. Numerous natural and synthetic sirtuin activating compounds that work by allosteric mechanisms to stimulate sirtuin activity, are shown to offer health benefits in rodents, primates, and thought to work the same way in humans [44]. Resveratrol, a polyphenol found in red grapes, first characterized for its antioxidant properties and subsequently for the activation of sirtuins has been reported in multiple studies to extend lifespan in model organisms including *D. melanogaster* and *C. elegans* [51, 52]. Although generally resveratrol is thought to have lifespan extending effects, some studies in recent years have also claimed that resveratrol showed no lifespan expansion effects in the same models [53]. In a previous study, no effect of resveratrol on *Daphnia* lifespan was observed using the short-lived ecotype (*D. pulex* TCO clone), but the overall fecundity was reduced [54]. The effect of resveratrol on Sir2 activity in treated *D. pulex* was not tested and it remains a possibility that the concentrations of resveratrol used in the study may not have affected Sir2 activity levels significantly. Although we have not examined the effects of Sir2 knockdown in *D. pulex*, our results in *D. pulicaria* suggest that a possible variation in the genotypes of the two isolates may reveal important and interesting modifiers of Sir2 dependent regulation.

Another documented and endogenously produced small molecule regulator of sirtuins is melatonin, a free radical scavenger [55-57]. Melatonin levels decline with age and it was reported recently that melatonin activates sirtuins, in addition to regulating circadian rhythms and acting as an anti-inflammatory agent [55-57]. It was reported that melatonin had no effect on *Daphnia magna* lifespan but attenuated the neck teeth formation and stress response in presence of fish kairomones [58]. Although no correlation of the observed effects on stress response or lack of effect on lifespan with Sir2 activity was tested in this study, it is certainly possible that the melatonin concentrations used may not have affected Sir2 activity.

Caloric restriction extends lifespan of several species and improves healthspan. Many of these benefits have been attributed mainly to the enhanced activity of Sir2 in yeast and invertebrates and Sirt1 in mammals [52, 59, 60]. This view was challenged by a study describing the failure to observe extension of lifespan in worms and flies transgenic for the corresponding Sir2 orthologs in a uniform genetic background [61]. Nevertheless, considering the conflicting evidence, the physiological roles of Sir2 in caloric restriction may be more complex than expected initially. Caloric restriction was not seen to extend lifespan in *D. pulex*, however, Sir2 expression or activity was not studied in this context [54]. Although this study did not indicate an extension in lifespan following caloric restriction in *Daphnia*, an earlier study demonstrated that caloric restriction does extend the lifespan in multiple species of *Daphnia* including *D. pulex* and *D. pulicaria* [31]. It could be interesting to study activity of Sir2 under various food levels in different isolates and species of *Daphnia* in future studies.

The decline in Sir2 activity in older organisms is often attributed to a decrease in the essential cofactor NAD+ [19, 25, 62]. It is possible that the decline we observe in older *Daphnia* is also due to a decline in NAD+ levels and this remains to be determined. Another interesting aspect of Sir2 activity regulation is the effect of NAM amidases [19, 20]. NAM is a byproduct of NAD+ conversion during Sirt1 catalysis and is also a noncompetitive inhibitor of Sirt1 [20]. Multiple studies have shown that overexpression of a NAM scavenger enzyme, NAM amidase, extends lifespan by released inhibition of Sirt1 [20].

Our results for the first time establish a functional role of Sir2 activity in regulating *Daphnia* life span and as it becomes an established model organism for research on aging and longevity, the analysis of *Daphnia* Sir2 will be at the forefront of research in this field.

## METHODS

### Cloning of *Daphnia* Sir2 ORF to verify expression of functional protein

Primers were designed to clone the *Daphnia pulicaria* Sir2 ORF using MacVector based on the published *D. pulex* genome (DappuDraft_290541: wFleaBase) [36]. We designed four primers such that the two PCR products would cover the entire ORF in two overlapping pieces, the “5′ half” (883 bp) and the “3′ half” (1065 bp) with an overlap of 46 bp (Figure 1B) between the two halves around an internal MscI restriction enzyme site. The primers were designed with a restriction site Nde1 for the 5′ end of the 5′half and BamHI for the 3′ end of the 3′half (sequence of primers is given in the next section). The PCR products were cloned into pGEMT-EZ (Promega) and the sequence of *D. pulicaria* Sir2 ORF was confirmed and submitted to GenBank (KT960973). We utilized the internal MscI restriction enzyme cut site to join each half of the Sir2 PCR product from their respective 5′half Sir2/pGEMT EZ and 3′half Sir2/pGEMT EZ constructs. A three-piece ligation was performed to join the two halves of the *Daphnia* Sir2 ORF thereby sub-cloning the full length ORF into the pBSIIKS+ (Stratagene) that contained an in-frame FLAG tag on the amino terminus of Sir2. A final sub-cloning in pcDNA3.1- (Invitrogen) resulted in the FLAG- Sir2/pcDNA 3.1- expression construct used for this study.

### Reverse transcriptase PCR

Total RNA was isolated from *D. pulicaria* using RNAzol B reagent (TelTest). cDNA was synthesized at using random hexamer primers, 1 μg total RNA, M-MuLV reverse transcriptase, 500 μM dNTPs, and RNase Inhibitor RNasin (Promega) as previously described [12]. For each PCR reaction, 2 μl of total cDNA and 50 pmoles each of the forward and reverse primers were used with the Promega GoTaq PCR kit. The following conditions were used for PCR: 95^0^ C for 5 minutes (initial denaturation), denaturation at 95^0^ C for 30 seconds, annealing at 56° C for 5′ half Sir2, 58^0^ C for 3′ half Sir2, or 59^0^ C for GAPDH for 30 seconds, extension at 72° C for 60 seconds. We ran 40 cycles for the initial amplification of *Daphnia* Sir2 5′ and 3′ halves for cloning, and 27 cycles for the analysis of sir2 mRNA knockdown in the RNAi experiment. The linear range was determined by varying cycle numbers and performing a densitometric analysis of the amplified product for the reverse transcriptase PCR in the RNAi experiment. Note the Sir2 PCR product shown in Figure 6B is the 3′ half of *Daphnia* Sir2 ORF. Primer sequences used were as follows:

**5′ half Sir2**

Forward:

5′CATATGACCATGGCCGACGAACAAGGCGAG3′

Reverse:

5′ATGTTCTGCGTGTAGTTGCG3′

**3′ half Sir2**

Forward:

5′CGACCGTTCTTTAAATTCGC3′

Reverse:

5′TCAATCCATCGCTTTCCTTTTTAC3′

**GAPDH**

Forward:

5′TTATCACCTCCTCAACTTC3′

Reverse:

5′CTTCTTCCTTCACTTCTCC3′

### Western blot analysis to confirm expression of *Daphnia* FLAG Sir2

Cos-1 cells were transfected with 100 ng FLAG-Sir2/pcDNA3.1- or control FLAG-TRBP/pc DNA3.1-. 24 hours post-transfection, Cos-1 cells were harvested, washed with 1 ml of cold PBS twice and lysed in RIPA Buffer (150 mM NaCl, 1.0% IGEPAL, 0.5% sodium deoxycholate, 0.1% SDS, 50 mM Tris, pH 8.0), the lysates were centrifuged at 13000 Xg for 5 minutes at 4°C and the supernatants were used as total protein extracts. Total protein concentrations in the extracts were determined using the BCA kit (Pierce). 50 μg of protein per sample was resolved by SDS-PAGE on an 8% polyacrylamide gel. The primary antibody used was FLAG M2 from Sigma at a dilution of 1:2000. ECL plus (Pierce) chemifluorescence detection was used to detect signals on a GE Typhoon LAS4000.

### *In vitro* translation of *Daphnia* Sir2

*Daphnia* Sir2 was *in vitro* translated using the TNT T7 coupled reticulocyte system (Promega). We incorporated S^35^ methionine during translation to radioactively label *Daphnia* Sir2. Following *in vitro* translation, an SDS-PAGE was analysis was performed with an 8 % polyacrylamide gel that confirmed the presence of a radioactively labeled protein the same size as *Daphnia* Sir2. *In vitro* translated protein was then used in the Sirt1 Activity Assay.

### Sir2 activity assay to verify deacetylase activity of *Daphnia* Sir2 and to examine changes with age

We assayed for the deacetylase activity of *D. pulicaria* Sir2 using a commercially available kit from Abcam (ab156065). The kit is designed to measure the activity of any sirtuin; however, we used the Sirt1/Sir2 specific inhibitor EX-527 to determine the relative contribution of Sir2 activity present in *Daphnia* samples isolated from 1, 4, and 8 week-old organisms as well as *in vitro* translated *Daphnia* Sir2. The assay is based upon a proprietary substrate, which contains a fluorophore coupled to an acetylated peptide that also contains a quencher. Upon deacetylation, the quencher is cleaved by a peptidase included in the kit, and the fluorophore fluoresces, which is measured using a plate reader. In general, the higher the amount of fluorescence emitted, the higher the amount of Sirt1/Sir2 activity. All assays were performed using an opaque clear bottomed 96 well plate (UVstar from Greiner). Ten *Daphnia,* with embryos removed, were homogenized in lysis buffer (10 mM Tris HCl (pH 7.5), 10 mM NaCl, 15 mM MgCl_2_, 250 mM sucrose, 0.5% NP-40 and 0.1 mM EGTA). The samples were incubated on ice for 15 minutes and spun through 4 ml sucrose cushion (30% sucrose, 10 mM Tris HCl (pH 7.5), 10 mM NaCl, 3 mM MgCl_2_) at 1300 X g for 10 minutes at 4°C. The supernatant was discarded and the nuclear pellet was resuspended in ice-cold solution of 10 mM Tris HCl and 10 mM NaCl and pelleted via centrifugation at 1,300 X g for 10 minutes at 4°C. The washed, pelleted nuclei were lysed in 50 μl of extraction buffer (50 mM Hepes-KOH, pH 7.5, 420 mM NaCl, 0.5 mM EDTA Na_2_, 0.1 mM EGTA, 10% glycerol) by gentle homogenization. The samples were incubated on ice for 30 minutes and centrifuged for 10 minutes at 20000 x g. The supernatant, the crude nuclear extract, was stored at −80°C until use in the assay. Protein concentration of the extracts was determined using the BCA kit (Pierce). The Sirt1 Activity Assay was performed as per the manufacturer's directions. We used human recombinant Sirt1 as a positive control and EX-527 to ensure Sirt1 specificity. All plates were read on a Molecular Devices Gemini EM Microplate reader with an excitation wavelength of 340 nm and emission wavelength of 460 nm for the duration of 30 minutes with reads occurring every minute.

### Mammalian cell viability assay to confirm *Daphnia* Sir2 function

Cos-1 cells were transfected with 500 ng *Daphnia* Flag-Sir2/pcDNA3.1- expression construct or 500 ng empty vector pcDNA3.1- using Effectene (Qiagen). 24h after transfection the cells were subjected to a severe heat shock for either 20 minutes or 30 minutes at 42^0^ C. The control cells (without heat shock) remained at 37^0^ C for the duration of the entire experiment with no changes in viability (data not shown). 24 hours following the heat shock, cell viability was determined via Trypan Blue viability assay. Trypan Blue staining was done by mixing 200 μl of trypsinized cell suspensions with 200 μl of Trypan Blue followed by an incubation at room temperature for 3 minutes. The cell mixture was mixed briefly and 20 μl of this suspension was loaded onto a hemacytometer slide and cell viability was determined by counting the blue cells (dead cells) and total cells visualized by a light microscope. The percentage viability was calculated by counting at least 300 cells in each sample.

### Luciferase reporter assays to demonstrate *Daphnia* Sir2's role in response to proteotoxic stress

Cos-1 cells were co-transfected with *Daphnia* Flag-Sir2/pcDNA3.1- expression construct along with 1 ng pRL null (Promega) and 250 ng pGL4.41 (Promega) using Effectene (Qiagen) as per the manufacturer's instructions. pGL4.41 has the firefly luciferase gene under the control of 4 tandem copies of a consensus mammalian heat shock element (HSE). 24h after transfection the cells were treated with 30 μM CdCl_2_ to induce proteotoxic stress. For treatment with CdCl_2_, the culture medium was removed and replaced with DMEM with charcoal stripped FBS containing 30 μM CdCl_2_. Six hours following the addition of CdCl_2_, the cell extracts were harvested and the luciferase activity was measured using the Promega dual luciferase reporter system as per the manufacturer's instructions. The firefly luciferase activity was normalized to the Renilla luciferase activity to account for differences in transfection efficiencies in different samples. Fold induction following cadmium chloride treatment was calculated for each set by dividing the normalized luciferase activity (firefly luciferase activity divided by the Renilla luciferase activity) in the cadmium treated samples by the normalized luciferase activity in untreated samples. The control represents the fold induction obtained with pGL4.41 reporter co-transfection with empty vector pcDNA3.1- following cadmium chloride treatment.

### Real time PCR

RNA was isolated from *D. pulicaria* at ages 1 week, 4 week, and 8 week using RNazol B (Teltest). cDNA was synthesized as described before using random hexamers [12]. We standardized our real time PCR reactions by performing real time PCRs with serial dilutions of cDNA to ensure appropriate reaction efficiencies. In order for primer sets to be used we set a threshold of 95% efficiency and both primer sets used were within this range (97% for Sirt1, 98% for GAPDH). All reactions were performed in triplicates in a total volume of 20 μl. Reactions included 4 μl cDNA, 250 nM Sirt1 or GAPDH primers, and SsoFast EvaGreen Supermix (BioRad). GAPDH was used for normalization and the primers were same as described by Scoville and Pfrender [63]. All reactions were run on a BioRad CFX96 Real Time System C1000 Thermal cycler machine with the following conditions: 95^0^ C for 30 seconds, 95^0^ C for 5 seconds, 52^0^ C for 5 seconds (the 95 C for 5 seconds and 52 C for 5 second steps are repeated for 40 cycles), 65^0^ C for 5 seconds, and then 95^0^ C for 5 seconds. The data was analyzed using the Bio-Rad CFX Manager Software and used the 2^−ΔΔCt^ method to compare Sir2 expression in 1, 4, and 8 week RNA samples. Note that 3 separate RNA isolations were used from 3 separate groups of *Daphnia* to serve as biological replicates. Primer sequences used were as follows:

**Real Time Amplicon Sir2**

Forward:

5′-GCAGCGAGGATGAAAATCTC-3′

Reverse:

5′-CTCCCAGTCATGATTGCTCA-3′

**GAPDH**

Forward:

5′-TTATCACCTCCTCAACTTC-3′

Reverse:

5′-CTTCTTCCTTCACTTCTCC-3′

### RNAi of Sir2

We created a dsRNA expression construct for the RNAi feeding regimen using the plasmid vector L4440 (AddGene #: 1654, originally a gift to Addgene from Dr. Andrew Fire). We sub-cloned the 5′ half of *Daphnia* Sir2 ORF in L4440 to express a Sir2 dsRNA of corresponding length (Fig. 5A). This construct would produce a dsRNA of 883 bp corresponding to the 52 half of Sir2 mRNA and thus will target the 52 half of the endogenous transcript. The Sir2/L4440 plasmid was transformed into BL21 (DE3) bacteria, which contain an integrated T7 RNA polymerase under the control of lacUV5 promoter. The bacteria were grown in Luria broth containing 2mM IPTG to induce the expression of the T7 RNA polymerase and consequent production of the Sir2 dsRNA as both strands of the Sir2 ORF are expressed from two opposing T7 promoters in L4440 plasmid. Bacterial cells from 1 ml of the overnight culture (OD_600_ = 2.6) were pelleted and resuspended in 1 ml of COMBO. 500 μl of this suspension was then added daily to the beakers containing the experimental *Daphnia*. For setting up the experiments, between 7 and 12 *Daphnia* neonates were collected and grouped per 100 ml of COMBO. Bacterial feed was administered each day, along with 20,000 cells/ml of the algae *Ankistrodesmus falcatus*. The death and reproductive output of *Daphnia* were recorded every day until all organisms had perished. Water was changed every other day to ensure no additional growth of bacteria was occurring in beakers and the amount of bacteria consumed was equal each day throughout the course of the experiment. We used two controls, a wild-type control that was not fed on bacteria and a dsRNA control that encodes GFP dsRNA (L4417, AddGene #: 1649, originally a gift to Addgene from Dr. Andrew Fire). GFP dsRNA serves as a control for any effect the presence of dsRNA can potentially exhibit, as there is not endogenous target for GFP dsRNA. To investigate if the knockdown of Sir2 was occurring properly, a separate set of *Daphnia* of the same age were fed a bacterial RNAi regimen for 10 days. After ten days, the *Daphnia* were sacrificed for further analysis via Reverse Transcriptase PCR.

### *Daphnia* viability assay following Heat Shock

To determine the ability of *Daphnia* to cope with proteotoxic stress (specifically heat shock), we standardized the temperature and duration of heat shock such that it elicited death after 24 hours of the heat shock. Ten *Daphnia* were placed per microcentrifuge tube in 1 ml of water and we had 3 replicates per temperature (total 30 Daphnia with 10 in each tube). The microcentrifuge tube was incubated in a heating block at the designated temperatures for the duration of the heat shock (20 or 30 minutes). Following the heat shock, the *Daphnia* were placed in 100 ml of COMBO and allowed to recover for 24 hours before analyzing percentage death. Death was determined by visually inspecting the *Daphnia* under a light microscope to ensure that there was no heartbeat (they have a transparent carapace that makes visual analysis of the heartbeat simple).

### Statistics

We performed several statistical tests in order to analyze our results. Either Student T-tests, Chi Square Analysis, Analysis of Variance (ANOVA) (followed by post hoc Tukey test) or nonparametric log rank tests (for survivorship data) were performed depending upon the data (Note: figure captions list which statistical test was performed for each data set). Each figure caption denotes p values as set forth by brackets and special characters. Note that our alpha level for multiple comparisons was p=0.05.

### *Daphnia* cultures

*Daphnia pulicaria* used in this study were clone 11 isolated from Lake Sixteen (42.564 N, 85.615 W) in Martin, Michigan, USA in 2008 and have since been cultured in the lab. No explicit permission is required to collect *Daphnia* from lakes that are public access in the state of Michigan. *Daphnia* are neither endangered nor protected. More details on the source population of *D. pulicaria* are available in Dudycha 2004 [32]. *Daphnia* were maintained at a temperature of 20^0^ C with a photoperiod of 12:12 L:D (12 hours of light followed by 12 hours of dark) within a Percival growth chamber. *Daphnia* were maintained at a concentration of 3 to 5 organisms per 250 mL beaker in 100 mL of COMBO except during RNAi feeding experiments (see below for specific details). *Daphnia* were cleared of young and transferred to a new beaker with fresh water on every alternate day and were fed every day with vitamin-supplemented algae *Ankistrodesmus falcatus* at a concentration of 20,000 cells/mL. To generate experimental groups, even-aged cohorts were created by placing neonates individually in 100 ml of COMBO medium [64], which is an artificial lakewater. Experimental animals were otherwise maintained as in the source cultures.
